# Breastfeeding in the workplace: Other employees' attitudes towards services for lactating mothers

**DOI:** 10.1186/1746-4358-3-25

**Published:** 2008-10-20

**Authors:** Kathryn Suyes, Sheryl W Abrahams, Miriam H Labbok

**Affiliations:** 1 State Child Fatality Review Team, Office of the Chief Medical Examiner of Virginia, Richmond, VA, USA; 2 Department of Maternal and Child Health, Gillings School of Global Public Health, The University of North Carolina at Chapel Hill, Chapel Hill, NC, USA; 3 Carolina Breastfeeding Institute, Department of Maternal and Child Health, Gillings School of Global Public Health, The University of North Carolina at Chapel Hill, Chapel Hill, USA

## Abstract

**Background:**

Workplace accommodations for breastfeeding mothers are an important step towards achieving United States Healthy People 2010 goals for continued breastfeeding. However, evidence suggests that some employers wishing to accommodate lactating mothers fear negative reactions from other workers.

**Methods:**

This study conducted in February 2007, used descriptive statistics and linear regression to assess attitudes towards workplace breastfeeding/milk expression among employees (n = 407) of a large U.S. corporation providing a wide variety of workplace accommodations for lactating mothers.

**Results:**

Overall, attitudes about the impact of breastfeeding on the work environment were favorable. Previous exposure to a co-worker who breastfed or expressed milk during the work day was associated with a positive attitude towards workplace breastfeeding, even after controlling for respondents' gender, length of employment and personal breastfeeding history.

**Conclusion:**

These preliminary findings suggest that lactation accommodations did not have negative repercussions for other employees, and that a corporate environment designed to enable and encourage continued breastfeeding does not endanger positive attitudes towards breastfeeding in other employees.

## Background

More than half of American mothers of children under the age of three years participate in the workforce [1]. The return to work postpartum often coincides with cessation of breastfeeding, suggesting that women employed outside the home face workplace-related challenges to continued lactation [2,3]. It follows that workplace accommodations for lactating mothers are important to achieve United States Healthy People 2010 objectives of 50% of mothers to breastfeed for 6 months and 25% breastfeeding for 1 year [4]. Such accommodations could range from adequate break time for milk expression and designated spaces for nursing/pumping, to coverage for lactation services within employee health plans and workplace crèches for on-site breastfeeding.

Fear of negative reactions from other employees has been reported as a barrier to employers' willingness to provide on-the-job accommodations for breastfeeding women [5]. However, few studies have examined how other employees actually react to such accommodations. The purpose of this study is to assess attitudes towards workplace breastfeeding and/or breast milk expression among employees of a large US corporation that provides a variety of on-site services for breastfeeding mothers.

## Methods

### Research aim

Specifically, we sought to test two hypotheses: 1) In a corporate environment in which comprehensive on-site services are provided to lactating mothers, the majority of employees will exhibit positive attitudes towards breastfeeding/milk expression in the workplace, and 2) Employees who had worked directly with a breastfeeding woman would exhibit more positive attitudes towards workplace breastfeeding than their peers who had not worked directly with a breastfeeding woman.

### Survey administration

An online multiple choice survey assessing breastfeeding-related experiences and attitudes was administered in February 2007 to employees at the headquarters of a privately owned corporation in the southeastern United States. A message inviting respondents to complete a Qualtrics^® ^survey for the chance to win prizes (e.g. restaurant gift certificates) was included in the monthly human resources bulletin sent to all company employees, including managers and executives. This corporation was chosen for study due to a reputation for "family-friendly" policies that include flexible work schedules, subsidized on-site child care, and paid family medical leave. The primarily professional positions offered at its headquarters include information systems, technical support, administrative assistance, sales, marketing and human resources. At the time of survey, forty-six percent of employees were female.

In addition to on-site lactation rooms, other services available to company employees and their spouses include on-site breastfeeding classes, lactation educators at an on-site health center, two on-site child care facilities, and discounts on breast pumps.

Nine questions were used to measure employee attitudes towards workplace breastfeeding (Table 1) [6]. These questions, developed by Bridges et al. to measure employer attitudes towards breastfeeding in the workplace [6], were used to capture respondents' perceptions of the impact of breastfeeding on the work environment. Responses were measured on a 5-point Likert scale, with selections ranging from "strongly agree" to "strongly disagree", and tabulated to create an Index of Breastfeeding Attitudes (IBA) score. Responses to those statements that expressed negative attitudes towards workplace breastfeeding were scored in reverse manner as those that expressed positive attitudes, to facilitate compilation of the index. Possible IBA scores ranged from 9 (most negative attitudes towards workplace breastfeeding) to 45 (most positive attitudes towards workplace breastfeeding).

**Table 1:** Index of Breastfeeding Attitudes (IBA)*

1. Formula fed babies are as healthy as babies who receive human milk.
2. Allowing women to breastfeed in the workplace will interfere with productivity.
3. Allowing women to breastfeed in the workplace will decrease the turnover rate.
4. Allowing women to breastfeed in the workplace will decrease absenteeism.
5. Allowing women to breastfeed in the workplace will improve morale of other employees.
6. Allowing women to breastfeed in the workplace will have a negative effect on the public image of the business.
7. Allowing women to breastfeed in the workplace will positively affect recruitment ability.
8. If a co-worker wanted to breastfeed her infant or express milk in the workplace, I would support it.
9. It is my responsibility to support mothers who combine breastfeeding with employment.

* Items 1, 2 and 6 scored in reverse.

### Statistical analyses

Data management was performed in Stata 10.0 (StataCorp, College Station, TX), and all statistical analyses were done in SAS 9.1 (SAS Institute Inc., Cary, NC). Descriptive statistics were tabulated, and t-tests and analysis of variance performed to examine differences in mean IBA score by background characteristics. Linear regression examined the association of having had a lactating co-worker with the IBA score. Potential confounders considered for inclusion in the regression model were respondent's age, gender, length of employment at the company, and whether or not the respondent had breastfed an infant. "Lactating co-worker" was defined as an affirmative response to the question "Have you ever worked with women who have breastfed or expressed milk during the work day?" The question as written did not specify whether the experience occurred with this or with a previous employer.

### IRB approval

This study was approved by the Institutional Review Board of the UNC School of Public Health.

## Results

### Respondent characteristics

Of the 4,069 employed at company headquarters, 407 completed the survey, for a response rate of 10%. Respondent characteristics are described in Table 2. Seventy-two percent of respondents were female, as compared to 46% of all company employees. The majority of respondents were 41 years of age and older, and had been employed by the company for 7 or more years (as compared to a median of 43 years of age and 9 or more years of employment for all employees). Because we received information on company demographic variables in summary form only, we were unable to conduct the necessary analyses to determine whether these differences were statistically significant.

**Table 2:** Respondent characteristics (n = 407)

	**n (%)**
**Gender**	
Female	293 (72.2)
Male	113 (27.8)

**Age**	
21–30 years	46 (11.3)
31–40 years	152 (37.3)
41–50 years	149 (36.6)
Over 50 years	60 (14.7)

**Length of employment at the company**	
Less than 1 year	24 (5.9)
1–6 years	167 (41.0)
7–15 years	143 (35.1)
More than 15 years	73 (17.9)

**Breastfeeding experience**	
Respondent had breastfed an infant (% of female respondents)	202 (68.9)
Spouse/partner had breastfed an infant	79 (19.0)
Respondent and/or spouse/partner had breastfed an infant while one was employed at the company	165 (40.5)
Had a co-worker who had breastfed and/or expressed milk during the work day	311 (76.4)

Sixty-nine percent of female respondents reported having breastfed an infant at some point in their lives (Table 2). Nineteen percent of all respondents reported that a spouse or partner had breastfed an infant. Forty percent of the sample reported that, during their employment at this company, they or a spouse/partner had breastfed an infant. Seventy-six percent reported having had a co-worker who breastfed or expressed milk during the work day.

### Attitudes towards workplace breastfeeding

Respondents' IBA scores ranged from 16–45, with mean and median scores equal to 35.1 (95% CI: 34.6, 35.6) and 35, respectively. An IBA score of 36 corresponds to a response of "agree" to each statement expressing positive attitudes towards workplace breastfeeding, and to a response of "disagree" to each statement expressing negative attitudes towards workplace breastfeeding. A score of 27, in comparison, corresponds to a neutral response of "neither agree nor disagree" to each of the attitude questions.

Mean IBA scores were higher among female respondents (mean score = 35.3, 95% CI: 34.7, 35.0) than among male respondents (mean score = 34.3; 95% CI: 33.4, 35.2); this difference did not achieve statistical significance (p = 0.08). Mean scores were somewhat higher among those respondents who reported that they or a spouse/partner had breastfed an infant while either was employed at the company (mean score = 36.4; 95% CI: 35.7, 37.0) compared to those who did not (mean score = 34.2; 95% CI: 33.5, 34.9) (p < 0.01). Scores were also higher among female respondents who reported having breastfed an infant (mean score = 36.6, 95% CI: 35.9, 37.2) than among female respondents with no personal breastfeeding history (mean score = 32.8; 95% CI: 31.6, 34.0) (p < 0.01). Mean IBA scores did not vary significantly by respondents' age category (p = 0.25).

Based on the results of linear regression, having had a co-worker who breastfed or expressed milk during the workday was associated with a 2.9-point increase in average IBA score (p < 0.01), before adjustment for other covariates.

In the full regression model (Table 3), having had a co-worker who breastfed or expressed milk was associated with a 2.4-point increase in average IBA score (p < 0.01), after controlling for gender, length of employment and whether the respondent had personally breastfed an infant at any point in her life. Age was removed from the full model after results indicated that it was not a significant predictor of IBA score.

**Table 3:** Adjusted mean change in Index of Breastfeeding Attitude score (n = 390)

**Variable**	**Parameter estimate**	**p value**
Reported lactating co-worker	2.43	< 0.01
Respondents' gender	-1.83	< 0.01
Respondents' length of employment	-0.56	< 0.01
Respondent has breastfed infant	3.62	< 0.01

Intercept = 36.69

## Discussion

The mean and median IBA scores indicate that, among employees of a company that provides a wide array of accommodations for lactating mothers, attitudes towards workplace breastfeeding/milk expression are on average positive. However, scores also reflect a wide distribution of responses. Though higher IBA scores were observed in the sample of respondents who had direct access to company lactation services (i.e. had been the parent of a breastfed child while employed at the company), average IBA scores among remaining employees also were positive.

Respondents reporting having had a co-worker who breastfed/expressed milk during the workday exhibited higher scores on the IBA than those who had not. This association remained after adjustment for confounders. This finding belies the concern that co-workers will react negatively upon witnessing the provision and use of special services for lactating mothers. Rather, one could infer from this that having first-hand experience with a co-worker breastfeeding in the context of a variety of lactation services/accommodations may actually engender more positive attitudes towards workplace breastfeeding.

Additional research is needed to further elucidate employees' reactions to specific components of the service package, to determine what specific benefits and disadvantages non-breastfeeding employees may perceive as arising from lactation accommodations for nursing mothers, and to examine employee attitudes in other geographical regions and other sectors, including blue collar industries and agricultural settings.

This study has several limitations. First, the survey's low response rate raises questions of respondent bias, namely, whether respondents who completed the survey did so out of prior interest in, or support for, breastfeeding. Due to anonymity of responses, the researchers had no way to determine which employees completed the survey, or to follow up with those who had not. Further, findings may be impacted by the facts that the survey respondents were more likely than non-respondents to be female, and the median age and length of employment were slightly lower among respondents than non-respondents.

When asked about breastfeeding co-workers, respondents were not asked to confirm that said co-worker was from this particular company. Thus it is possible that some respondents may have responded based on experiences with a different employer who may not have made the same accommodations for breastfeeding mothers. The extent to which this may have changed outcomes is unclear.

Finally, the population from which the sample is drawn reflects a highly educated group of individuals who may have chosen their employer based on agreement with its work-life programs and family-friendly workplace policies. Therefore, results may not be generalizable to all industries or other populations of employees.

## Conclusion

Despite these limitations, these findings contribute to our understanding of the important issue of breastfeeding support in the workplace and highlight areas that deserve additional study. In summary, our findings do not support the previously published perception that a corporate environment designed to enable and encourage continued breastfeeding will engender overall negative attitudes in other employees.

## Competing interests

The authors declare that they have no competing interests.

## Authors' contributions

KS designed the study, created and administered the survey, and edited the manuscript. SA analyzed the data and drafted the manuscript. ML assisted in conceptualization of the study, design of the survey, analysis planning, and editing of the manuscript.

## References

[B1] Bureau of Labor Statistics, US Department of Labor (2006). Women in the Labor Force – A Databook Washington, DC.

[B2] Visness CM, Kennedy KI (1997). Maternal employment and breast-feeding: findings from the 1988 National Maternal and Infant Health Survey. Am J Public Health.

[B3] Hendricks K, Briefel R, Novak T, Ziegler P (2006). Maternal and child characteristics associated with infant and toddler feeding practices. J Am Diet Assoc.

[B4] US Department of Health and Human Services Healthy People 2010 (2^nd ^ed). http://www.healthypeople.gov/Document/HTML/Volume2/16MICH.htm#_Toc494699668.

[B5] Brown CA, Poag S, Kasprzycki C (2001). Exploring large employers' and small employers' knowledge, attitudes, and practices on breastfeeding support in the workplace. J Hum Lact.

[B6] Bridges CB, Frank DI, Curtin J (1997). Employer attitudes toward breastfeeding in the workplace. J Hum Lact.

